# Computational Ways to Enhance Protein Inhibitor Design

**DOI:** 10.3389/fmolb.2020.607323

**Published:** 2021-02-03

**Authors:** Robert L. Jernigan, Kannan Sankar, Kejue Jia, Eshel Faraggi, Andrzej Kloczkowski

**Affiliations:** ^1^Roy J. Carver Department of Biochemistry, Biophysics and Molecular Biology, Iowa State University, Ames, IA, United States; ^2^Research and Information Systems, LLC, Indianapolis, IN, United States; ^3^Department of Physics, Indiana University Purdue University Indianapolis, Indianapolis, IN, United States; ^4^Battelle Center for Mathematical Medicine, Nationwide Children's Hospital, Columbus, OH, United States; ^5^Department of Pediatrics, The Ohio State University, Columbus, OH, United States

**Keywords:** protein design, peptide design, computational design, protein ensemble, protein potentials

## Abstract

Two new computational approaches are described to aid in the design of new peptide-based drugs by evaluating ensembles of protein structures from their dynamics and through the assessing of structures using empirical contact potential. These approaches build on the concept that conformational variability can aid in the binding process and, for disordered proteins, can even facilitate the binding of more diverse ligands. This latter consideration indicates that such a design process should be less restrictive so that multiple inhibitors might be effective. The example chosen here focuses on proteins/peptides that bind to hemagglutinin (HA) to block the large-scale conformational change for activation. Variability in the conformations is considered from sets of experimental structures, or as an alternative, from their simple computed dynamics; the set of designe peptides/small proteins from the David Baker lab designed to bind to hemagglutinin, is the large set considered and is assessed with the new empirical contact potentials.

## Introduction

Influenza infection is a widespread cause of major medical concern because of rapid viral evolution, which causes both occasional pandemics and, more frequently, health problems almost every year. It has been estimated that the annual outbreaks by influenza A and B viruses over the past 100 years have had an even greater impact than all other past pandemics combined (Wilson et al., 1981; Bullough et al., 1994; Bizebard et al., 1995). The extremely high mutation rate of the virus means that any given vaccine soon becomes outdated. Thus, vaccination offers limited protection, especially when facing the highly virulent nature and rapid evolution of influenza (Chen et al., 1999). Although some effective anti-influenza drugs have been developed, drug resistance usually appears rapidly.

Hemagglutinin (HA) is a major surface glycoprotein of this virus that is involved in four of the most important aspects of influenza infection: (a) it is the target of antibodies that neutralize infectivity, (b) it undergoes antigenic drift to escape neutralization, (c) it binds to cell-surface receptors to initiate infection, and (d) it mediates the fusion of viral and host membranes essential for viral entry. The large-scale conformational changes in HA are critical for the steps in which the virus inserts itself into the host cells by fusing to the host membrane, and the residues involved in this process are highly conserved across different types and subtypes during antigenic drift. These residues can serve as important targets for developing broad-reacting antiviral inhibitors (Jiang et al., 1993; Wild et al., 1994; Chan et al., 1998; Skehel and Wiley, 1998). Based on a set of crystal structures of the HA-antibody complex showing the conformational changes to HA during the essential activation steps, David Baker and his colleagues designed a novel HA inhibitor for Group 1 of type A virus (Fleishman et al., 2011).

**Figure 1:**
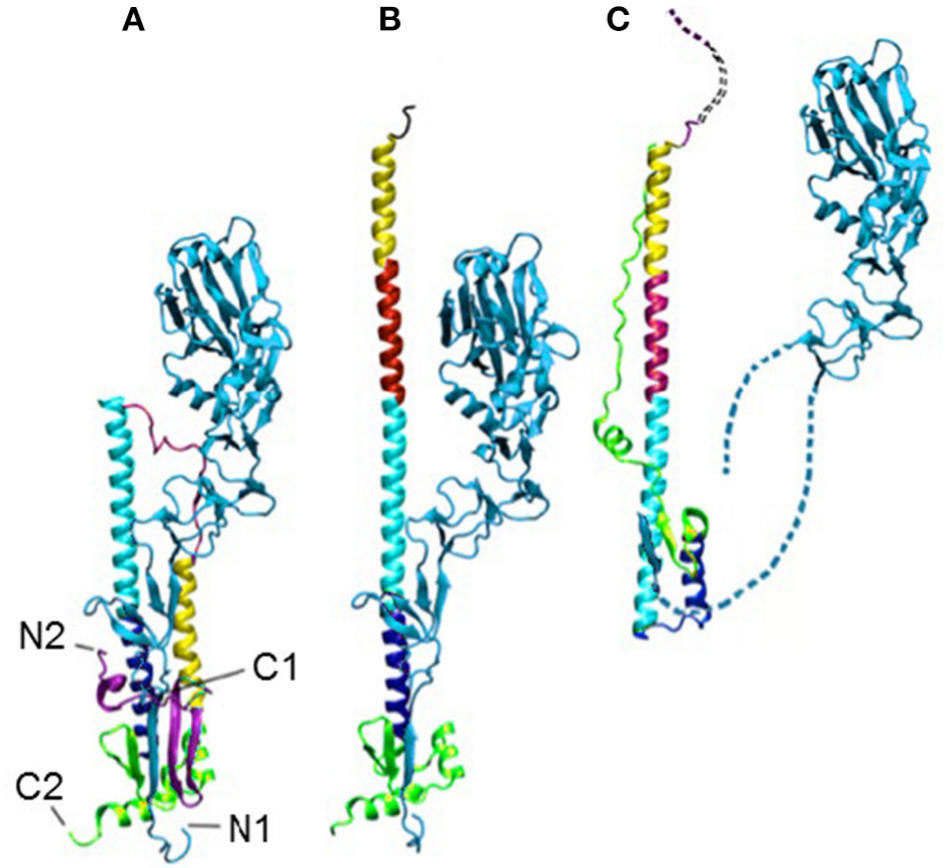
The HA in the pre- **(A)**, intermediate **(B)**, and post-fusion **(C)** states. The termini of HA1 (light blue) and HA2 are labeled as N1, C1, N2, and C2, respectively, in **(A)**. The dotted lines in **(C)** indicate unresolved regions. The structures have all been aligned on the cyan helix, which is the only region in that domain that does not change during the transition.

Influenza HA is a homo-trimeric protein where each monomer contains two disulfide-bonded polypeptides, HA1 and HA2. HA1 is responsible for attaching to host cell-surface receptors, and HA2 mediates the fusion of the influenza envelope with the endosomal membrane thus allowing the entry of influenza RNA into the host cell. The pre- (Wilson et al., 1981) and post-fusion structures (Bizebard et al., 1995) of HA1 are essentially the same, while those of HA2 (Wilson et al., 1981; Bullough et al., 1994; Chen et al., 1999) are drastically different (see Figure 1).

The structural change in HA2 includes a partial unfolding of the long α-helix into a loop (dark blue) and the folding of an inter-helix loop (in red) into a part of the long α-helix, thus delivering both N- (blue) and C-terminal (pink) fragments to the same end of the molecule upon the fusion of viral and endosomal membranes.

The protein gp41 of HIV-1 is the membrane fusion protein, similar to HA2 of HA (Skehel and Wiley, 1998). In that case, peptides derived from the C-terminal region of gp41 corresponding to the outer-layer helices, referred to as C-peptides, were found to inhibit HIV-1 infection with IC_50_ in the nanomolar range (Jiang et al., 1993; Wild et al., 1994; Chan et al., 1998). C-peptides are believed to act by binding to the exposed surface of the N-terminal central three-helical bundle in a transient pre-fusion gp41 intermediate, thereby blocking membrane fusion. One such L-peptide, T-20/Enfuvirtide with 36 residues, was approved previously as a drug by the Food and Drug Administration (FDA) (FDA Notifications, 2003); it showed high efficacy in suppressing resistant HIV-1 strains. Moreover, efforts to target a prominent pocket on the surface of the central three-helical bundle have led to the discovery of small, cyclic D-peptides that inhibit HIV-1 infection, thereby validating the pocket as a potential target for small-molecule HIV-1 fusion inhibitors (Eckert et al., 1999).

**Figure 2:**
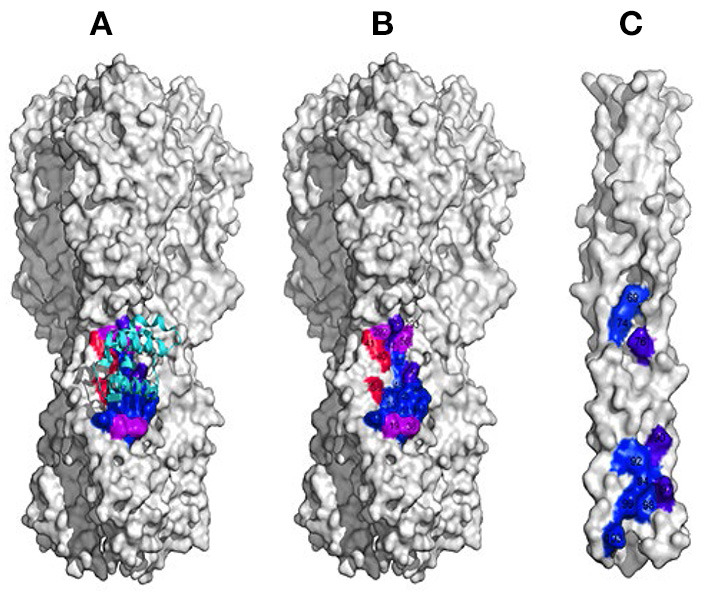
Interfaces on HA that could likely be targeted in inhibitor design. **(A)** The interface conserved among Group 1 influenza A virus (in colors). **(B)** The interface conserved among all influenza A and B viruses. **(C)** The conserved patch on the surface of the central helical bundle at low-pH on HA.

To evade host antibody recognition, the HA protein on the surface of influenza virus, primarily on the globular domain, must constantly mutate. This interferes in important ways with any vaccine and reduces the vaccine's efficiency and useful lifetime. However, no matter how much the influenza virus mutates, it must maintain the ability to induce membrane fusion to ensure its propagation. Thus, the stem domain that is primarily responsible for inducing membrane fusion is the most conserved. Ian Wilson's group identified antibodies that broadly neutralize influenza A virus Group 1 (Ekiert et al., 2009) (Figure 2A), Group 2 (Murphy and Webster, 2001), Group 1 and 2 (Ekiert et al., 2012), and influenza type A and B viruses (Dreyfus et al., 2012) (see Figure 2B). All these antibodies recognize epitopes located in the stem domain. David Baker's group designed small proteins against influenza A virus Group 1 (Fleishman et al., 2011) (Figure 2A). In addition, they identified a conserved patch on the surface of the central helical bundle in the low-pH post-fusion state (Figure 2C). These three interfaces may all serve as useful targets for developing inhibitors against influenza virus.

Molecular recognition in general and protein-protein interactions in particular are essential in almost every aspect of biological function. Moreover, proteins that bind other proteins with high affinity and high specificity have numerous applications for diagnostics and therapeutics. Currently, antibodies are by far the most commonly used proteins for both detection and therapeutic intervention. However, antibodies are large proteins that are expensive to produce and difficult to deliver. Thus, it would be important progress for biomedicine to be able to design novel protein-binding modules at will.

The set of 88 proteins that were designed and tested by Baker et al. provides an excellent test set for use in the present study. Below we consider the dynamics of the structure in two different ways, from a set of experimental structures and from computed dynamics. Then we apply new knowledge-based free energies to rank the different designs, specifically predicting which designs are likely to bind. Baker and colleagues were not able to do this without experimental testing. These are empirical-free energy contact potentials developed by Jernigan, Kloczkowski, and Faraggi that have proven to be highly successfully in blind-tests at past CASP experiments. In the present paper, we aim to make some suggestions for new ways to sample conformations of a target protein and how to assess the designed structures.

## Multiple Experimental Structures Capture the Important Functional Motions Within a Hemagglutinin Structure Set

The 43 structures of hemagglutinin listed in Table 1 were collected from the PDB with a BLAST search, retaining only those structures present as trimeric complexes of the HA1 and HA2 subunits. The individual subunits were extracted separately and aligned. This yields a total of 129 structures of the HA1 + HA2 monomers that were superimposed onto the central structure (PDB: 1 mqm) using the Combinatorial Extension (CE) algorithm, and these have a continuous distribution of RMSDs from 0 to 3.3 Å.

**Table 1:** The PDB identifiers of the 43 structures of hemagglutinin used here for extracting dynamics.

1HGD	2HMG	3FKU	4BGZ	4KPQ
1MQL	2IBX	3HMG	4BH1	4KPS
1MQM	2WR7	3LZG	4DJ6	5HMG
1MQN	2WRB	3M5G	4EDB	
1RD8	2WRD	3M6S	4F23	
1RUY	2WRE	3S11	4F3Z	
1RUZ	2WRF	3SM5	4FIU	
1RV0	2WRG	3UBE	4GXX	
1RVX	2WRH	3VUN	4JTX	
2FK0	3EYM	3ZTJ	4KDM	

**Figure 3:**
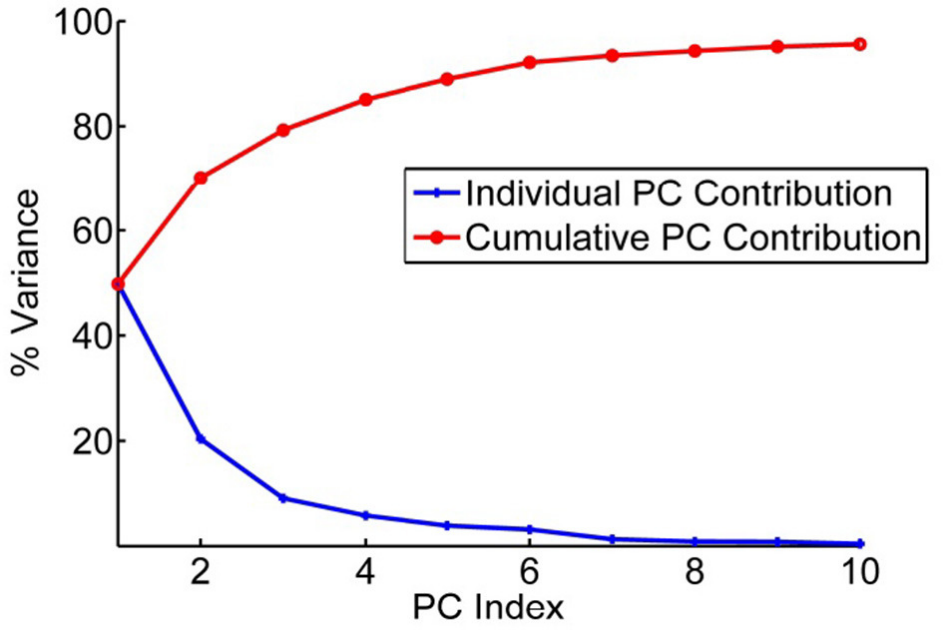
Principal component contributions to the total motions of hemagglutinin. Percent of variance explained by each individual PC is shown in blue and the cumulative contribution of each PC to the total variance/motion in red. The first 5 PCs account for 90% of the total motions present in the set of 43 structures.

After these structures have been superimposed, the covariances for all pairs of positions were computed. Then Principal Component Analysis is performed on this dataset. The input is the set of all of the structures in the set (Teodoro et al., 2002, 2003). From these data, the average position of each point in the reference structure is computed as < *x*_*i*_>, and the covariances for each pair of points, *i* and *j*, was computed according to *c*_*ij*_ = 〈(*x*_*i*_ − 〈*x*_*i*_〉)(*x*_*j*_ − 〈*x*_*j*_〉)〉, where brackets < > indicate averages over the set of structures. The covariance matrix *C* can be decomposed as *C* = *PΔP*^*T*^, where the eigenvectors *P* represent the principal components (PCs) and the eigenvalues are the elements of the diagonal matrix Δ. The eigenvalues are sorted in order. Each eigenvalue is directly proportional to the amount of the total variance it captures. The results of this analysis are shown in Figure 3 for the set of coarse-grained hemagglutinins, which shows how truly limited the characteristic motions are within the structure set. Clearly, it does not require many of these characteristic motions to capture nearly all of the overall motions.

## Characterization of the Global Motions in Hemagglutinins

**Figure 4:**
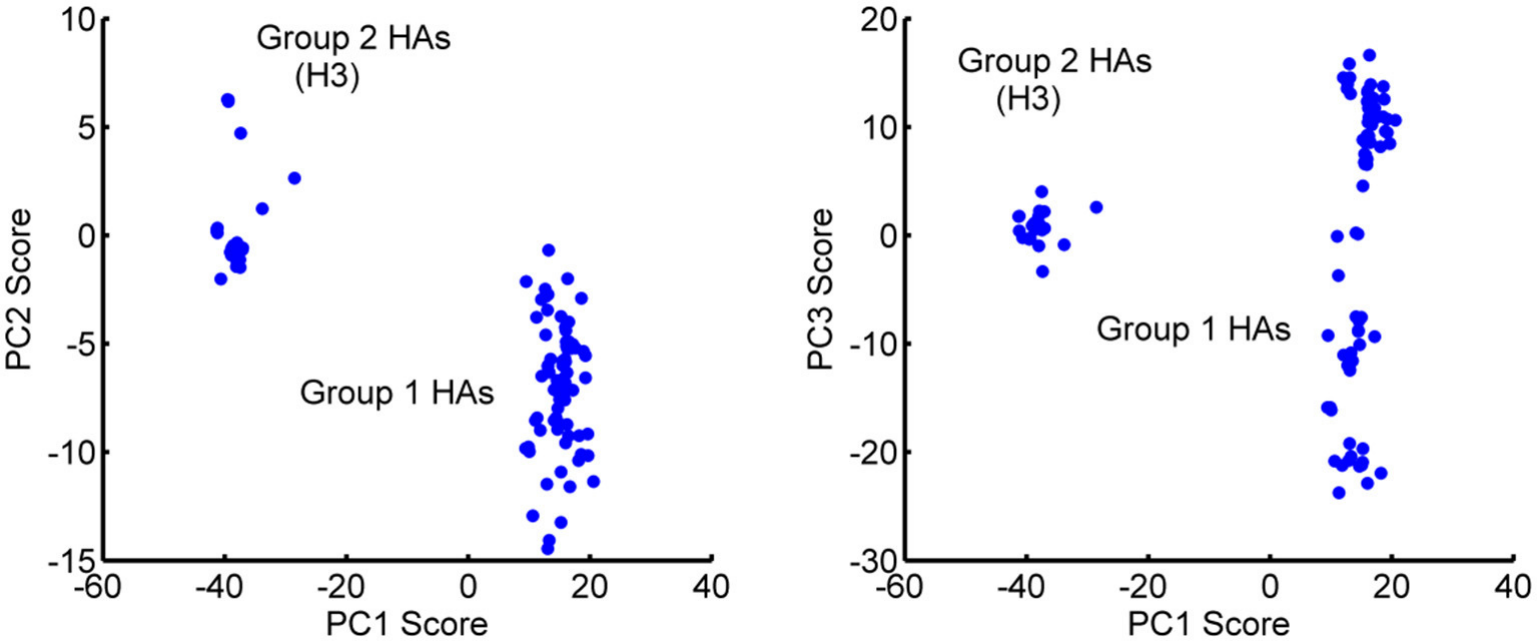
Distribution of the 129 HA monomeric structures projected onto the first 3 PCs. **(Left)** PC2-PC1 space and **(Right)** PC3-PC1 space. PC1 separates the Group 1 and Group 2 hemagglutinins into different clusters. Outliers H13 and H16 have been eliminated from the figure for the sake of clarity. PC1 has a major gap between the two groups of clustered structures. PC3 appears to be populated in two somewhat similar clusters, with Group 1 showing a particularly wide range of PC3 values.

Based on their sequences, HAs have been subdivided into two main groups: Group 1 (H1, H2, H5, H6, H8, H9, H11, H12, H13, and H16) and Group 2 (H3, H4, H7, H10, H14, and H15) (Air, 1981). Interestingly, the first three PCs separately cluster into these two major groups, with minor exceptions. The distribution of the experimental structures over the PCs are shown in Figure 4 for pairs of PCs. This distinctive clustering can be seen clearly.

**Figure 5:**
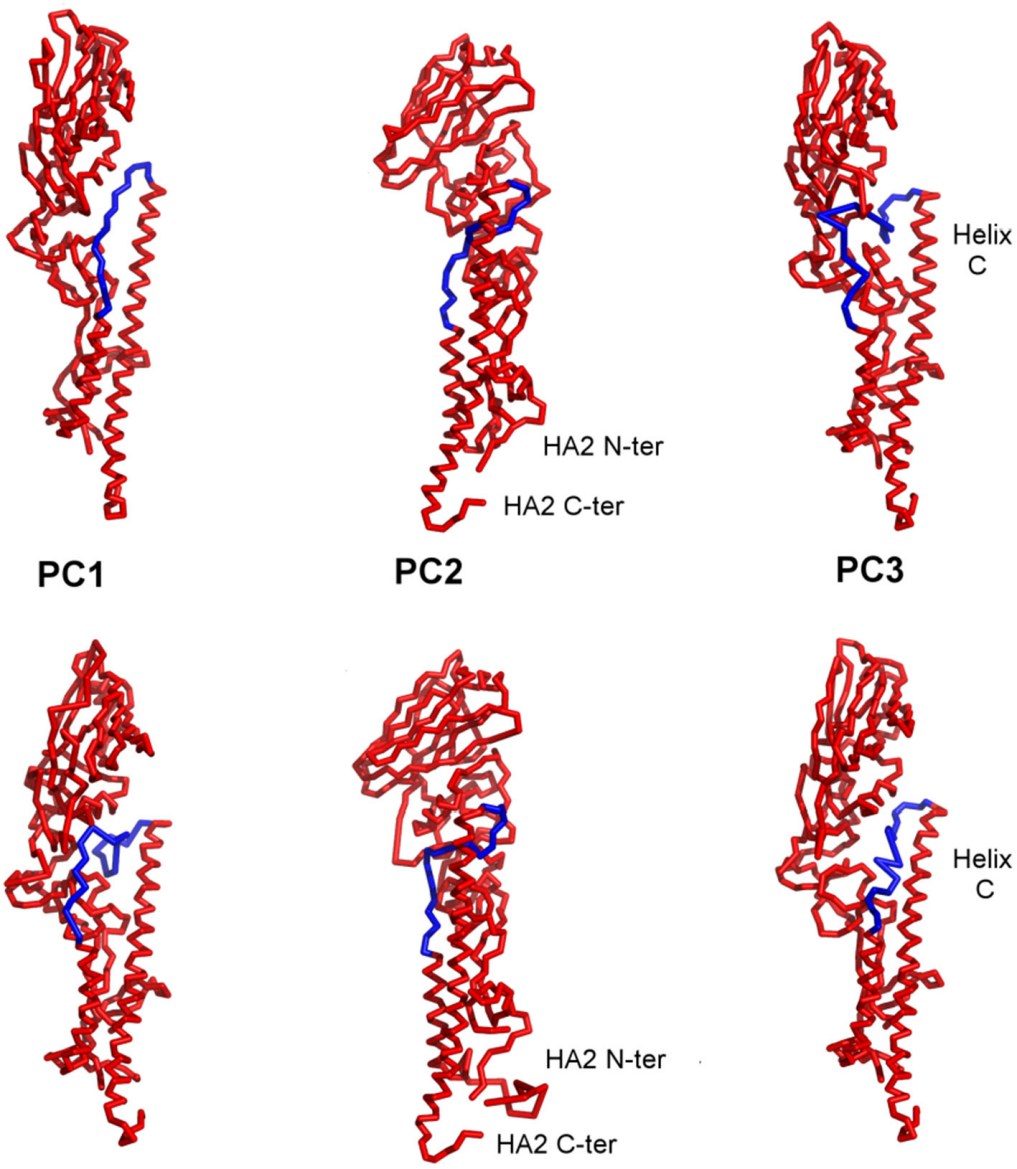
Visualization of the first three PC motions on the structures of HA. The two structures shown in each column are two extreme conformations representative of the changes indicated in each individual PC. PCs 1, 2, and 3 can be identified as winding and unwinding of the C-terminal, central, and N-terminal parts of the B-loop (blue) into a helix. PC2 captures the hinge bending of the structure between the head and stem regions as well as movement of the N-terminus of HA2. The blue highlighted segments indicate the parts of the structure exhibiting a broad range of conformations.

Different conformations can bind to different partners and thus include dynamics in the process that will improve the probability of success in computational protein design. When the PCs are visualized on the structures, it can be seen that the first three PCs primarily represent motions in the B-loop (blue) that are involved in the large-scale transition. PC1, PC2, and PC3 can be interpreted as primarily involving conformations changes in the C-terminus, the central, and N-terminus parts of the B-loop (see Figure 5). Interestingly, the B-loop is a region with a strong tendency to form a coiled-coil and is implicated in the formation of the pre-hairpin intermediate in the “spring loaded mechanism” of HA action (Carr and Kim, 1993; Xu and Wilson, 2011). The PC3 motion also clearly demonstrates the shift in the loop necessary for it to position itself at the top of helix C. In addition, PC2 captures a hinge motion in the head of HA with respect to the stem as well as well as a motion at the N-terminus of HA2 (fusion peptide) that is subsequently exposed for insertion into the membrane during fusion. These computed structures show a high level of variability of conformations particularly for the B-loop, which relate well to the known conformational transition, even though the full extent of motions is not shown in Figure 5. As shown in Table 2, these PCs provide a useful representation of changes present in the ensemble of structures.

**Table 2:** Cumulative overlaps between computed ANM modes and PCs from the set of experimental hemagglutinin structures.

	**CO**		
	**3 Modes**	**6 Modes**	**20 Modes**
PC1	0.60	**0.66**	**0.71**
PC2	**0.50**	**0.57**	**0.65**
PC3	0.40	0.44	**0.60**

*Values above 0.5 are in bold*.

## Anisotropic Network Models (ANM) Can Substitute, of Insufficient Numbers of Experimental Structures are Available

Elastic Network Models of proteins, such as the Gaussian Network Model (GNM) and Anisotropic Network Models (ANM) of proteins as developed by Tirion (1996), Bahar, Erman, and Jernigan (Bahar and Jernigan, 1994, 1998; Bahar et al., 1997a; Demirel et al., 1998; Atilgan et al., 2001; Doruker et al., 2002a,b; Doruker and Jernigan, 2003; Sen et al., 2006), computationally yield information about protein fluctuation dynamics, the directions of motions of the residues and atoms around their equilibrium positions. This information has already been used by Bahar, Jernigan, Kloczkowski, and many others with significant success (Bahar and Jernigan, 1994; Keskin et al., 2002a,b; Isin et al., 2012) to explain functional motions and mechanisms in proteins, nucleic acids, and large biological assemblies, such as the ribosome. ANM could be used as an alternative to calculate the normal modes from a single structure when insufficient numbers of experimental structures or structures having sufficient variability are not available to perform PC analysis, then normal modes from the elastic network models could also be used to compute entropies (Zimmermann et al., 2012) (But, as we show below, contact entropies are simpler and provide significant gains). In ANM, the potential energy *V* is a function of the displacement vector *D* of each point in the structure $V=\frac{\gamma }{2}DHD^{T}$, where γ is the spring constant for all closely interacting points in a structure (here we used a cutoff distance of 13 Å between alpha-carbons for coarse-grained models retaining only C^α^ atoms) to establish the spring connections between residues), and *H* is the Hessian matrix containing the second derivatives of the energy, with respect to each of the coordinates x, y, z. For a structure with *n* residues, the Hessian matrix *H* contains *n* × *n* super-elements each of size 3 × 3. The Hessian matrix *H* can be decomposed (Atilgan et al., 2001) as *H* = *MΛM*^*T*^, where Λ is a diagonal matrix comprising the eigenvalues with the eigenvectors forming the columns of the matrix *M*. This decomposition generates 3*n*-6 normal modes (the first six modes account for the rigid body translations and rotations of the system) reflecting the vibrational fluctuations, so singular value decomposition is utilized.

## Comparing Directions of Motions Using Overlaps

The alignment between the directions of a given experimental PC and a given computed normal mode can be measured by comparing the directions of motion in their overlap, as defined by Tama and Sanejouand (2001): $O_{ij}=\frac{\left|P_{i}\cdot M_{j}\right|}{\left\|P_{i}\|\|M_{j}\right\|}$, where *P*_*i*_ is the *i*th PC for and *M*_*j*_ is the *j*th normal mode. A perfect match yields an overlap value of 1, meaning these motions are in the same direction. We also define the cumulative overlap (CO) between the first *k* vectors *M*_j_ and *P*_*i*_ as $CO\left(k\right)={\left(\overset{k}{\underset{j=1}{\sum }}O_{ij}^{2}\right)}^{\frac{1}{2}}$.

The high overlaps between the two methods ensures the reliability of the computed dynamics. The 1st, 2nd, and 3rd PCs have good overlaps of 0.57, 0.43, and 0.34 with the 3rd, 2nd, and 1st individual modes, respectively. We compare the first three PC's from the X-ray set with the first 20 normal modes from the elastic network models, and these are relatively high between all three PCs of the X-ray hemagglutinin and the set of normal modes for the computed normal modes (see Table 2).

## Strategies for Generating and Ranking an Ensemble of Structures and Identifying a Structure Module Targeted for Inhibitor Design

**Figure 6:**
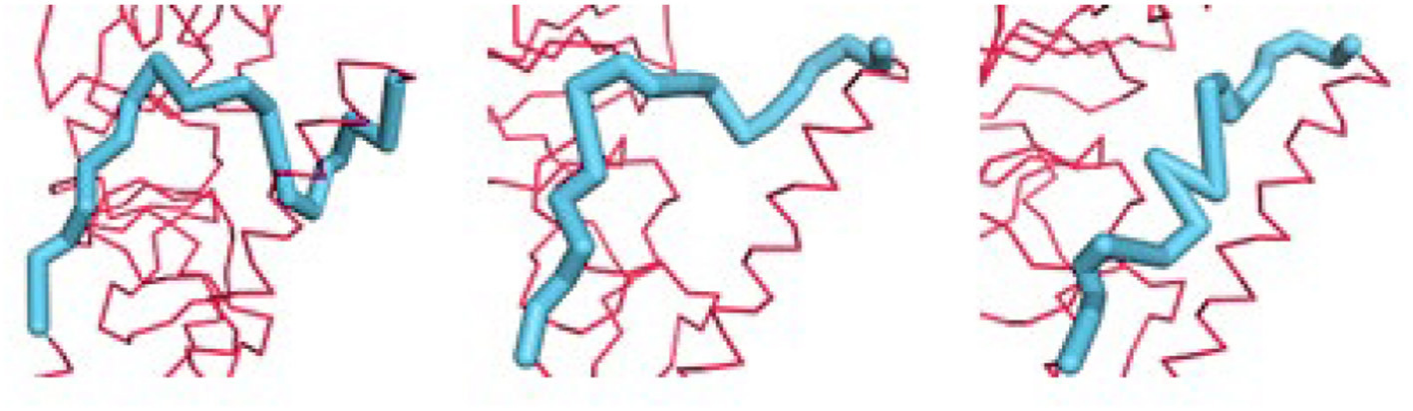
Examples of the diversity of conformations from the first 3 PCs for the B loop (blue) of hemagglutinin. The PCs can be used to generate an ensemble of conformations. Each of three shows a conformation generated from one PC. The motions showed that this loop is the most flexible part of the structure and possesses an extremely diverse set of conformations.

Identifying the most conformationally variable part of the structure is the aim here. These are the parts of a structure that should be the most useful to use for inhibitor design. These parts can be identified simply by computing the changes in all internal distances over the ensemble. Examples of such potential binding parts to target have been extracted from the ensemble of sampled conformations for HA generated by utilizing combinations of the first several PCs (Figure 6). This highly variable segment should be susceptible to binding by a broader range of ligands.

## Assessing Peptide/Protein Designs With New Empirical Contact Potentials

Here we present new strategies for the assessment of bound ligand structures by taking as our target the designed small proteins from David Baker and his colleagues that were targeted to bind hemagglutinin (Fleishman et al., 2011; Fleishman and Baker, 2012). This provides an interesting relatively large dataset, which we can use to test our assessment method. The Baker designs, originating from small, monomeric proteins in the PDB having between 80 and 250 residues, were targeted against a hydrophobic region on the “stem” of hemagglutinin. Of the 88 designs that they tested, only two were reported to have detectable binding affinity for hemagglutinin (this affinity was subsequently improved in rounds of randomization and selection).

### Four-Body Coarse-Grained Contact Potentials (Feng et al., 2007, 2010)

Four-body potentials were developed by Kloczkowski and Jernigan to account for the cooperative interactions in proteins; they take into account the coarse-grained contact interactions together with the extent of solvent exposure, and thus provide a more detailed and more cooperative representation of protein interaction energies than do pairwise potentials. Capturing this cooperativity is considered to be critical for evaluating densely packed protein structures. These potentials are highly empirical and are based simply on the observed frequency of occurrences of different types of amino acids in closely interacting quartets of amino acid types within a large set of protein structures. We have found that these four-body contact potentials can discriminate well between native structures and partially unfolded or deliberately misfolded structures. These have also included short-range backbone energies (Bahar et al., 1997b). We tested these optimized potentials at CASP9 as the prediction group 4_BODY_POTENTIALS from Iowa State University. There were 110 other human prediction groups participating in CASP9 competition, and 140 prediction servers. According to Nick Grishin, the assessor of free modeling techniques at CASP9, 4_BODY_POTENTIALS was one of most successful groups in free modeling at that time, ranking third according to the averaged z_score_ both for best models and top models. Free modeling is the most difficult and most challenge in protein structure prediction, when the sequence of the protein has only a low sequence similarity in comparison to any known protein structures. This success at CASP9 demonstrates clearly that the cooperative multibody interactions are an appropriate tool for assessing predicted structures, and we apply them here to Baker's hemagglutinin inhibitor structures. Later we added in electrostatic interactions, and these were tested at the subsequent CASP10.

### Including Entropies in the Inhibitor Assessments

The Elastic Network Models (ENM) have proven themselves to be highly useful in representing the global motions for a wide variety of diverse protein structures (Bahar and Jernigan, 1997, 1998, 1999; Bahar et al., 1997a,b,c; Bahar et al., 1998, 1999; Demirel et al., 1998; Keskin et al., 1998, 2000, 2002a,b; Jernigan et al., 1999, 2000, 2008; Atilgan et al., 2001; Bahar and Rader, 2005; Sen et al., 2006; Jernigan and Kloczkowski, 2007; Yang et al., 2007, 2008, 2009; Zhu and Hummer, 2010; Bakan et al., 2011; Karaca and Bonvin, 2011; May and Brooks, 2011; Peng and Head-Gordon, 2011; Uyar et al., 2011; Wieninger et al., 2011; Zheng, 2011; Zheng and Auerbach, 2011; Zimmermann et al., 2011a,b; Duttmann et al., 2012; Gniewek et al., 2012; Isin et al., 2012; Martin et al., 2012; Ruvinsky et al., 2012; Globisch et al., 2013; Kim et al., 2013; Sanejouand, 2013; Dasgupta et al., 2014). Since they have proven to be so successful in capturing the most important motions of protein structures, it is reasonable to expect that they should also be able to estimate the conformational entropies of structures. We employ the Elastic Network Model to compute the motions of protein structures, and then these motions are then used directly to approximate the entropy of a conformation (Zimmermann et al., 2011c, 2012). We previously (Zimmermann et al., 2011c) used vibrational entropies based on the frequencies of the normal modes, but more recently have found significant gains by utilizing the mean square fluctuations computed from the ENM as a direct measure of entropy: $\Delta S\propto \Gamma^{-1}=\overset{N}{\underset{i=2}{\sum }}\frac{1}{\lambda_{i}}\left(Q_{i}Q_{i}^{T}\right)$, where *Q* is a normal mode vector, λ the corresponding square frequency, Γ the system's Hessian, and Γ^−1^ its pseudo-inverse. We obtain the Free Energy changes from ΔG = ΔE – TΔS by simply combining the four-body potential with the ENM-based entropy (Zimmermann et al., 2012). The excellent blind-tested performance of our method in CASP experiments shows that our methodology is an outstanding tool for assessing protein designs, such as the ones from Baker's hemagglutinin inhibitor designs.

## These New Free Energies Successfully Select Native-Like Poses in Protein-Protein Docking

**Figure 7:**
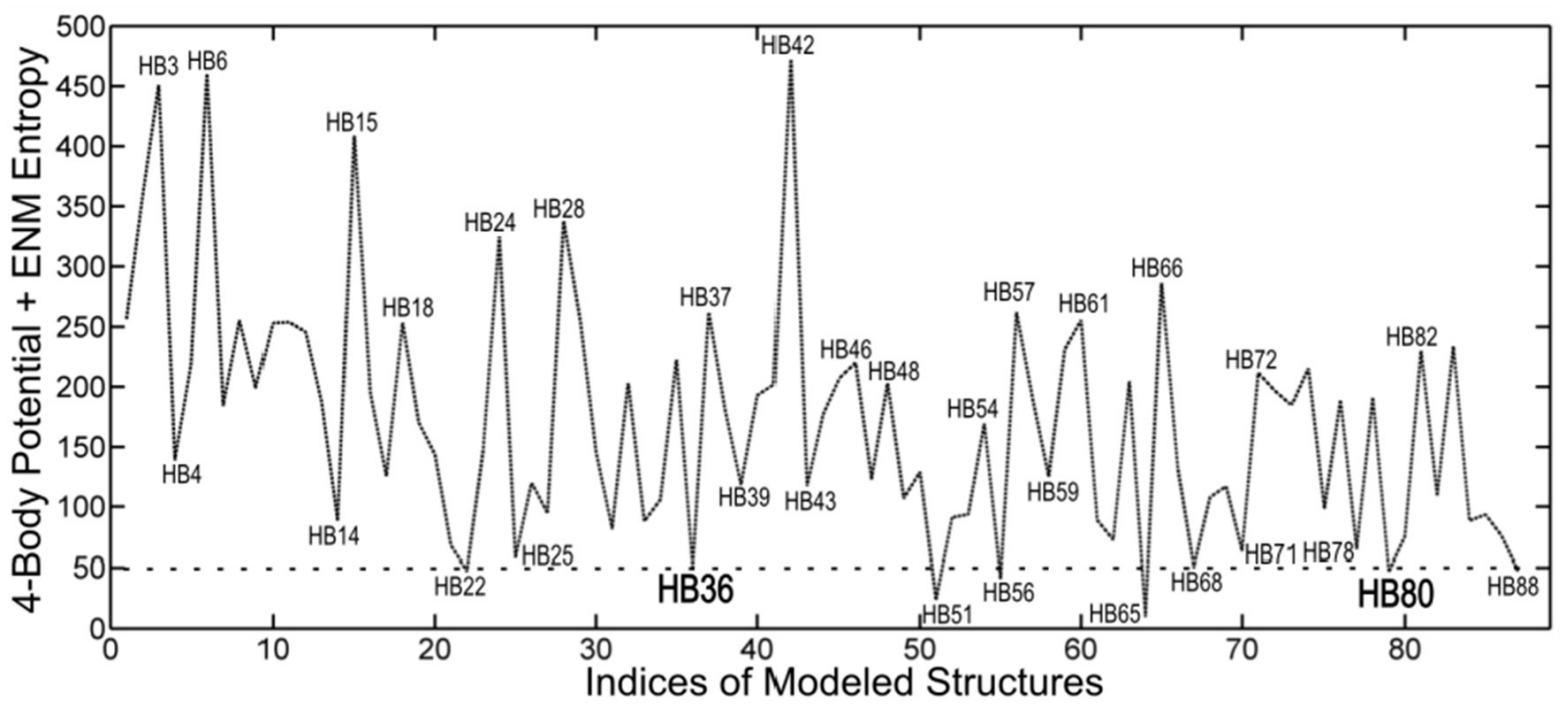
Ranking by coarse-grained free energies of inhibitor proteins designed against hemagglutinin. Free energies are given on the ordinate axis (arbitrary scale), and the different structures (from the pdb) are indicated along the abscissa. The eight top-ranked structures with favorable free energies can be seen to be HB22, HB36, HB51, HB56, HB65, HB68, HB80, and HB88. This demonstrates the utility of the coarse-grained free energies to computationally screen for favorable structures. The two structures HB36 and HB80 were experimentally shown to be functional.

We have applied this method to the set of 89 inhibitor proteins designed against hemagglutinin by David Baker's group, and we find that it provides a useful screen for that set of structures. Structures having the lowest energies indicate stable favorable conformations. However, stable structures are not always functional. In this case, we tested a set of eight structures at local minimums of the energy landscape ranked by their energies. From these, it was reported that two of them were found to be functional (see Figure 7).

## Discussion

We have outlined a simple new way to use protein dynamics for peptide/protein design studies. This approach serves to identify those specific regions in the structure having particularly wide-ranging conformational variability, which could be of particular importance for targeting computational design efforts. Specifically, the highly variable segments should be able to bind to a particularly wide range of diverse ligands. Such variable conformations are well-known to be important for the promiscuous binding exhibited by disordered proteins and using this approach should have some advantage. Using such more localized protein targets might be an important new approach for targeted computational design. Another advantage of this is that more exhaustive computations can be carried out for smaller targets.

Application of the potentials described above to assess structural designs would allow ranking of sets of designed inhibitor proteins. The differences in rankings should allow to conclude the extent to which the large-scale backbone fluctuations identified in the dynamics could be utilized in the design process. This would require a significantly larger effort than has been presented here. Of course, the potentials themselves are empirical and could be modified to reflect the data from the experimental studies on the designed molecules for the specific class of targeted protein, which is one of the major advantages of the adaptability of the empirical potentials in any particular application.

Our approach can be extended by detailed analysis of allosteric sites that are important for drug design. Most drugs are designed to bind directly to the primary active sites, called orthosteric sites, to inhibit or modify the function of the protein. Binding of a drug to the active site prevents binding to a virus or other disease-related agent and most drugs are designed to fit into the primary active sites. However, adverse side effects of a drug may occur because many enzymes or receptors with related functions may have similarities in their active sites.

A new approach to drug design is based on secondary binding site effects. In this approach, small molecule drugs are designed to bind at secondary binding sites called allosteric sites (Tsai and Nussinov, 2014; Dokholyan, 2016; Guarnera and Berezovsky, 2016, 2020; Schueler-Furman and Wodak, 2016; Wodak et al., 2019; Zhang et al., 2020). A potential drug—an allosteric modulator binds to an allosteric site and remotely modifies the conformation of the primary binding site of the protein. Allosteric sites are controlled by intrinsic protein dynamics, and the approach proposed here could also be applied to these allosteric sites.

## Data Availability Statement

Publicly available datasets were analyzed in this study. This data can be found at: protein data bank.

## Author Contributions

RJ, KS, KJ, and AK conceptualized, carried out the research, and wrote the paper. EF engaged in discussions about this research. All authors contributed to the article and approved the submitted version.

## Conflict of Interest

The authors declare that the research was conducted in the absence of any commercial or financial relationships that could be construed as a potential conflict of interest.
